# Machine Learning Analysis of Essential Oils from Cuban Plants: Potential Activity against Protozoa Parasites

**DOI:** 10.3390/molecules27041366

**Published:** 2022-02-17

**Authors:** Renata Priscila Barros de Menezes, Luciana Scotti, Marcus Tullius Scotti, Jesús García, Rosalia González, Lianet Monzote, William N. Setzer

**Affiliations:** 1 Post-Graduate Program in Natural and Synthetic Bioactive Products, Federal University of Paraíba, João Pessoa 58051-900, Brazil; renatabarros@ltf.ufpb.br (R.P.B.d.M.); luciana.scotti@gmail.com (L.S.); 2 Pharmacy Department, Faculty of Natural and Exact Sciences, University of Oriente, Santiago de Cuba 90500, Cuba; jgadi1990@gmail.com; 3 Toxicology and Biomedicine Centre (TOXIMED), University of Medical Science, Santiago de Cuba 90500, Cuba; rosaliagonzalez.9110@gmail.com; 4 Parasitology Department, Institute of Tropical Medicine “Pedro Kouri”, Havana 10400, Cuba; 5 Aromatic Plant Research Center, 230 N 1200 E, Suite 100, Lehi, UT 84043, USA; 6 Department of Chemistry, University of Alabama in Huntsville, Huntsville, AL 35899, USA

**Keywords:** essential oil, Cuban plants, machine learning analysis, antiprotozoal activity

## Abstract

Essential oils (EOs) are a mixture of chemical compounds with a long history of use in food, cosmetics, perfumes, agricultural and pharmaceuticals industries. The main object of this study was to find chemical patterns between 45 EOs and antiprotozoal activity (antiplasmodial, antileishmanial and antitrypanosomal), using different machine learning algorithms. In the analyses, 45 samples of EOs were included, using unsupervised Self-Organizing Maps (SOM) and supervised Random Forest (RF) methodologies. In the generated map, the hit rate was higher than 70% and the results demonstrate that it is possible find chemical patterns using a supervised and unsupervised machine learning approach. A total of 20 compounds were identified (19 are terpenes and one sulfur-containing compound), which was compared with literature reports. These models can be used to investigate and screen for bioactivity of EOs that have antiprotozoal activity more effectively and with less time and financial cost.

## 1. Introduction

An essential oil (EO) is a concentrated plant secondary metabolite composed of a mixture of volatile chemical compounds [1], with a long history of use in food, cosmetics, perfumes, agricultural and pharmaceuticals industries [2]. In the last decade, almost 5000 articles related to uses of EOs have been published, with a positive increment of more than 7% per year [3]. In this scenario, the scientific, economic, and biological importance of EOs is growing as alternatives to synthetic compounds commonly used in industry [4,5,6].

In particular, numerous studies demonstrated the wide pharmacological spectrum of EOs, including: antimicrobial [7,8], antifungal [9], antiparasitic [10], antiviral [10,11], insecticidal [12], anticarcinogenic [13,14,15,16], immunomodulatory [17], anti-inflammatory and antioxidant [14]. Nevertheless, the chemical composition of obtained EOs can unfortunately be different depending on the chosen method, geographical origins, the season, the type of soil and the agricultural conditions in which plants have grown. Thus, the same plant could produce different EO chemical composition profiles and therefore display different biological effects [3,8,14]. In this sense, some approaches have been used, such as the ‘chemotype concept’ with the aim to discern the bioactivity of EOs based on chemical profile. However, more complex questions have emerged due to the high complexity in the chemical composition of EOs and interaction among constituents. Then, the role of each single constituent and synergistic/antagonist effects among components remain unclear in many potential EOs. During the last decade, computational studies using EOs have been reported in the continuous search for new therapeutic drugs or lead compounds. In particular, machine learning analysis has been used to identify new structures from EOs with potential antibacterial [18] and antiviral [3] activities.

Recently, potential EOs from Cuban plants was reviewed, which a variable number of components (terpenes, aliphatic derivatives, sulfur-containing compound, phenylpropanoids, alkaloids and amine-type compounds) and different biological activities (antiprotozoal, antibacterial, antifungal, anticancer, anthelmintic, larvicidal and insecticidal) were identified. However, correlation of potentialities of these EOs with chemical entities could not be linked due to scarce number of pure compounds that were tested and experiments related to synergistic/antagonistic effects were not found [19]. Then, in line with those previous studies (which common compounds were identified from different EOs with multiple biological effects), herein an extensive study of those EOs from Cuban plants is reported, with the main goal of finding chemical patterns between 45 samples and antiprotozoal activity, using unsupervised and supervised machine learning, which are Self-Organizing Maps (SOM) and Random Forest (RF), respectively.

Kohonen’s self-organizing maps [20] are neural networks of the unsupervised type, based on the functioning principle of the central nervous system of animals [21,22,23,24]. SOM has competitive learning, with only one output neuron or local group of neurons that provides the final response to a current input signal. The data presented in the input neuron are mapped to the defined space of neurons with iterations and weight adjustments in a typically two-dimensional array (Kohonen map). The most common way to demonstrate the similarity in SOM is through the Euclidean distance between the vectors and the input data vector [21,22,23,24].

The U-matrix, created by Vesanto and collaborators [24], is used to visualize the SOM. This matrix allows the visualization and discrimination of the groups generated in the SOM, from the Euclidean distances. This degree of similarity is plotted in the third dimension generating a 3-D relief surface, in this way the clusters are represented in the form of “depressions”, “valleys”, and “peaks”. The “depressions” and “valleys” of the relief surface of the U-matrix represent neurons belonging to the same cluster, while neurons that have a great distance from the adjacent neuron are represented by “peaks”, they are cluster-discriminating neurons.

RF is an algorithm that will create several decision trees at random, thus obtaining a forest where each tree will be used in the result. It is a robust and complex algorithm, which can lead to a higher computational cost compared to others. A decision tree establishes rules for decision making, that is, the algorithm will generate a structure like a flowchart with “nodes” where a condition will be checked and if met, the flow follows one branch, otherwise, it follows another, always leading to the nearest “node” where further decision-making will take place, until the end of the tree. Thus, given a training set, the algorithm will analyze the data and look for the best conditions and where to insert each data into the flow [25,26,27,28].

In the literature, previous studies using these methods have been applied to EOs, which have been useful to select antiviral and low toxic samples [3], antibiofilm formation by *Staphylococcus aureus* [16,29], *S. epidermidis* [16] and *Pseudomonas aeruginosa* [18,30], as different biological activities such as antiviral, anthelminthic, anti-inflammatory, anticancer, antioxidant, antimicrobial, antifungal and cytotoxic activity [31]. However, the application on EOs with antiprotozoal activity has been scarcely documented. The study performed herein, demonstrated how multidisciplinary applications involving machine learning could represent a valuable tool in predicting the bioactive component in complex mixtures.

## 2. Results and Discussion

**Figure 1:**
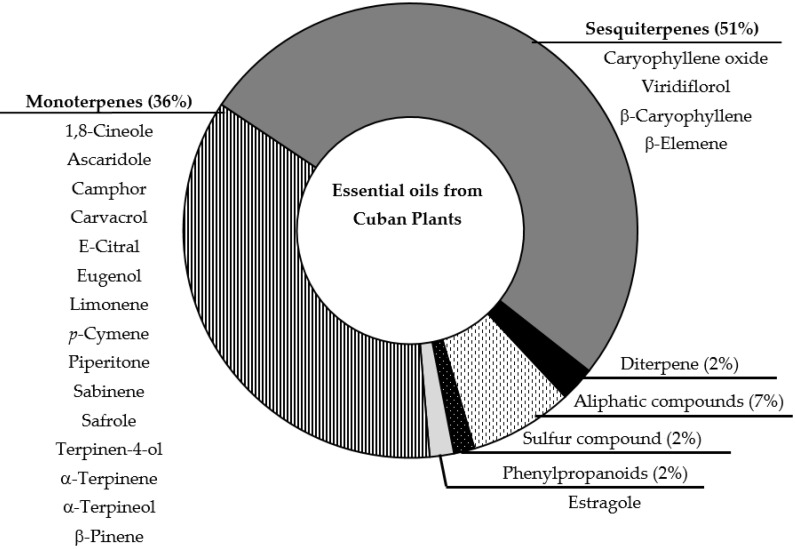
Schematic representation of chemical composition of analyzed EOs. Graphic represent the distribution of total components described for EOs; while list of compounds corresponds to main compounds identified in the EOs.

As previously reported, the analyses of 45 samples of EOs were included, which were obtained from 16 families, 33 species, and 408 different identified compounds [19]. Figure 1 represents the major compound class, as the components identified as main compounds in the different studied EOs previously reported [17].

The dataset of 45 EOs was analyzed to find a chemical pattern between the EOs and antiprotozoal activity, including three activities: antiplasmodial, antileishmanial and antitrypanosomal. The analysis started with the use of the SOM, where the chemical composition of the 45 EOs was used as information to find patterns with the antiparasitic activity. Among them, 21 had some of the analyzed activities (antiplasmodial, antileishmanial and antitrypanosomal), with median inhibitory concentrations (IC_50_) in in-vitro cultures < 100 μg/mL. The remaining EOs (24) had no antiprotozoal activity or activity had not been reported.

In the generated map, the hit rate was higher than 84%. The SOM validation was then performed using the 5-fold external cross-validation technique [32,33]; this means that the entire dataset is partitioned five times into a modeling set (training set) including 80% of the compounds in the set, and the external cross-validation data set, comprising the remaining 20% of the compounds in the data set. After this, only the modeling set is used to build the models and then the models are validated with the external cross-validation technique. In this sense, the dataset was subdivided into five training groups and five test groups, always keeping the ratio between active and not reported EOs. The validation results are described in Table 1.

Analyzing Table 1, we see that the hit rate for true positive rate (EOs active) and true negative rate (EOs that did not display antiprotozoal activity or had not been reported) both in training sets and in test sets were higher than 0.7, showing that the SOM model is robust. Model accuracy assessment gives information about the overall performance of the model, indicating the overall hit rate. The hit rate is the rate that evaluates how well the model correctly classified the EOs. Accuracy values vary between 0 and 1. Models with accuracy rate closer to 1 represents the higher model’s hit rate; while an accuracy rate equal to or greater than 0.7 is considered models of optimal performance [23,27].

The SOM managed to find a chemical pattern between the chemical composition of EOs and antiprotozoal activity. In parallel, we chose to check if this chemical pattern is also found by using a supervised algorithm, known as RF. The RF model was generated using the 5-fold external cross-validation technique [32,33]; this means that the entire data set is partitioned five times into a modeling set (training set) including 80% of the compounds the set, and the external cross validation data set, comprising the remaining 20% of the compounds the data set. After this, only the modeling set is used to build the models and then the models are validated with the external cross validation technique. Its performance was evaluated through the statistics such as specificity, sensitivity, which obtained satisfactory values that corroborate the accuracy of the superior model, at 70%. The performances can be observed in Table 2, these parameters are an average between the five models. During the creation of the model, we also observed the domain of applicability to ensure that the samples tested were within the chemical space of each model.

In Table 3, we can see the accuracy and the global hit of both models and show that the chemical same pattern could be obtained use an unsupervised (SOM) and supervised (RF) machine learning. In both analyses, an accuracy rates higher than 0.7 were appreciated.

**Figure 2:**
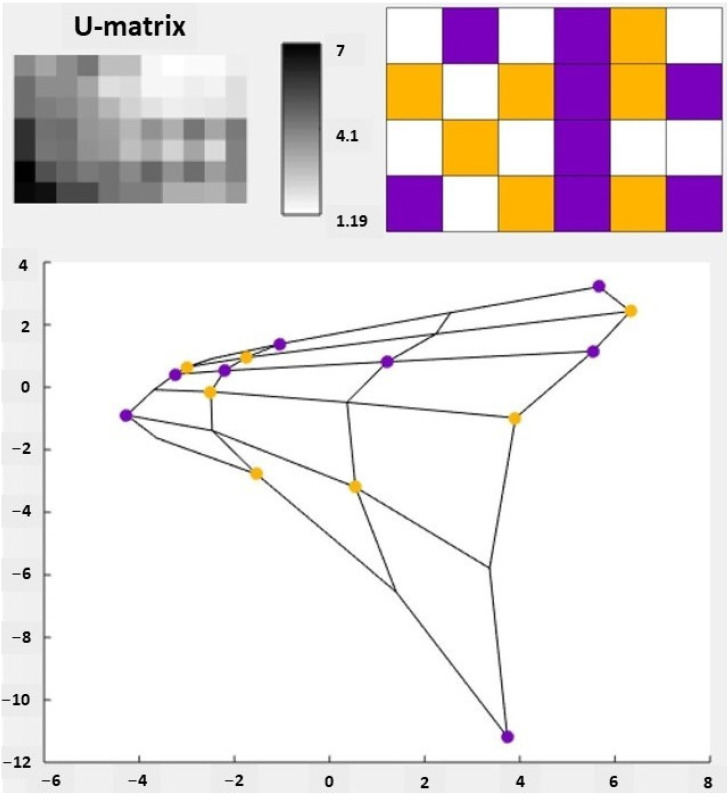
Visualization of the self-organizing map (SOM) of essential oil (EO) data. In the upper corner we have the U-matrix. The left U-matrix does not identify the activities of the EOs while the right U-matrix identifies those activities by color: active is violet and yellow represents samples with no antiprotozoal activity or that have not been reported. The values shown on the scale between the two U-matrices represent the values of the percent of molecules present in the EOs, varying between 1.19 and 7. These values were used to group the EOs by activities. At the bottom, we have the principal component analysis (PCA) projection of the SOM measured by its two eigenvectors with higher eigenvalues. The activities were plotted using the same identification colors as the U-matrix.

Figure 2 shows the U-matrix of the SOM, i.e., the visual analysis of the SOM. The U-matrix is constructed by measuring the Euclidean distance in the vector space between adjacent neurons [21,24,34]. It is possible to normalize the distances to be represented by colors or in shades of gray [21,24]. What is represented in the U-Matrix are the clusters mapped by the SOM and not the individual samples.

Forty-five EOs were used for the SOM analysis. After mapping the SOM, the 45 EOs were correctly grouped into active and inactive (EOs that do not display antiprotozoal activity or have not been reported). There was also the separation of groups of greater similarity and difference between them, taking into account the chemical composition of the EOs, which were approximated or distanced in the SOM. Thus, in the U-matrix, each square represents a group of EOs that are organized both by activity and chemical similarity, with the purple ones relating to active EOs and the yellow ones to inactive EOs.

It is also worth noting that the U-matrix is a visual representation of the topological mapping of the SOM, in this way, the white squares are valleys that separate the clusters that were generated.

It is also possible to observe in Figure 2 the principal component analysis (PCA) graph, which was generated from the correlation matrix of the EOs dataset used in the generation of the SOM. PCA is used to reduce the dimensionality of the data and allow a better visualization of the clusters, since it allows representing the input data as linear combinations of their projections [23]. The PCA performed in this study has an explained variance of 25.47%, that is, using only two variables it is possible to explain a quarter of the entire variance.

While in the U-matrix we have the white squares representing valleys that distance the clusters, in the PCA graph neighboring cartographic units are connected by lines to make the map view clearer and more defined [23].

**Figure 3:**
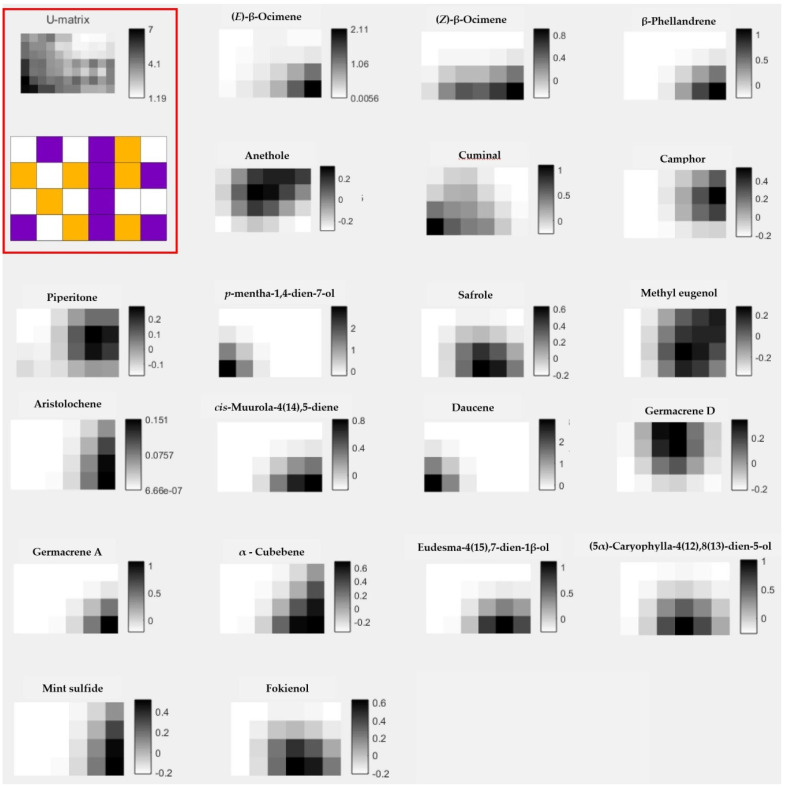
The most significant molecules for the EOs by activity. In the upper left we have the U-matrix of the self-organizing map generated in the study, with the upper U-matrix not identifying the tribes and the lower U-matrix identifying the tribes by color; active is violet and yellow represents samples with no antiprotozoal activity or that have not been reported.

After the general analysis with the 45 EOs, the SOM was constructed considering the chemical patterns of each sample. The most significant molecules for the chemical pattern separation of active and not reported EOs obtained with SOM Toolbox tool are shown in Figure 3. In this sense, 20 compounds present in the EOs were associated with at least one of these three biological activities, of which 19 are terpenes (10 monoterpenes and 9 sesquiterpenes) and one sulfur-containing compound. As is evident, a high predominance of terpene-type compounds was observed. Previously, the role of terpene compounds has been reviewed, suggesting the promising therapeutic value against protozoa parasites [35,36,37].

The identification of the most significant molecules is made by observing the region in the U-matrix of active EOs. Once the region was identified, we observed the most expressive molecules in that region. For example, when analyzing the U-matrix, we observe that in the lower right corner, there is a region in purple color, indicating a region of active EOs. Following the analysis, we will observe which molecules are most representative of that region; thus, we have the molecules (*E*)-β-ocimene, (*Z*)-β-ocimene and β-phellandrene. Note, in Figure 3, that the individual matrices of these molecules indicate their greater presence in the lower right region, the region of active EOs.

In a general comparison of listed components between Figure 1 and Figure 3, note that only three compounds match as major component of EOs and as significant molecules generated by SOM strategy: camphor, piperitone and safrole. In general, pharmacological studies of EOs suggest that major identified components could be responsible for the biological activity. However, some studies did not correlate the main compound with the antiprotozoal effect [38,39,40]. Thus, using the present model, we selected molecules present in EOs that can influence in the antiprotozoal activity of studied EOs, and could suggest other EOs based in the complete chemical composition and not only in the major components. In addition, it is interesting to specify that in the used data, camphor was identified in 5 samples with concentrations between 0.1 to 17.1%, piperitone was present in 7 samples ranging from 0.1 to 23.7%, and safrole was documented in 3 samples from 1.6 to 71.8% [17]. In regard to antiprotozoal activity, analysis of the samples with these compounds with concentrations higher than 5%, we note that, for example, camphor was reported in the EOs from *Piper aduncum* L. and *Piper aduncum* var. *ossanum* (C.DC.) Trel. that showed antiplasmodial, antileishmanial and antitrypanosomal activity, as well as piperitone. Safrole, in contrast, was identified in *Piper auritum* Kunt as major compound and displayed antileishmanial activity [17]. These examples could corroborate the observed results from the SOM analysis and probably could highlight *Piper* as a promising genus to study antiprotozoal properties, related with the main compounds or synergism resulting from the presence of these components in this genus. In fact, antileishmanial potentialities of the *Piper* genus was recently reviewed [41].

A quick literature search in Pubmed Electronic Database was carried out with 20 identified compounds. The most important results confirm that: β-ocimene and safrole have shown activity against *Trypanosoma brucei* with IC_50_ values of 1.1 μg/mL [42] and 18.4 μg/mL [43], respectively; while methyleugenol had an IC_50_ of 5.7 μg/mL against *Plasmodium falciparum* [44]. However, it was noted that several of the identified compounds were not evaluated against these protozoa parasites, which could be addressed in further screening assays.

Nevertheless, several studies in the literature have already confirmed the antiparasitic action of EOs with identified compounds obtained from plants in other geographical locations, which is summarized in Table 4 together with results of EOs from Cuban plants (supplementary material). The higher number of reports from Cuban and other EOs was found for camphor. For example, antiprotozoal activity was evaluated for EOs from Cuban plants against *Plasmodium falciparum, Leishmania* spp. and *Trypanosoma* spp. from *Alpinia zerumbet* (Pers.) B. L. Burtt & R. M. Smith [45], *Piper aduncum* L. [46] and *Piper ossanum* (C.DC.) Trel [47]; while the rest of EOs displayed activity only against kinetoplastid parasites from *Alpinia speciosa* K. Schum. [39], *Artemisia absinthium* L. [42], *Piper cubeba* L. [48], and *Thymus hirtus* sp. *algeriensis* Boiss. et Reut [30], which camphor proved to be one of the major substances in all included samples.

However, although in the literature, piperitone was only found in an EO from Benin with antitrypanosomal and antiplasmodial activity [49], in Cuban samples, it was found in higher concentrations of EOs (19 to 24%) that showed a broad spectrum of antiprotozoal effects mainly from *Piper* species [46,47]. In contrast, a diverse number of studies from worldwide plants, EOs with germacrene D and with antikinetoplastid activity correlated with antiplasmodial activity shown by Cuban EOs with this compound [50].

**Table 4:** In vitro antiprotozoal profile of Cuban and according literature review of EOs that present identified compounds in this study (previous shown in Figure 3).

Compound	Country	Plant	Compound %	Targeted Protozoa (Result)	Ref.
(*E*)-β-Ocimene	Brazil	*Annona vepretorum* Mart.	6.8%	*Trypanosoma cruzi* (IC_50_ = 32 μg/mL)	[51]
		*Syzygium cumini* (L.) Skeels.	11.7%	*Leishmania amazonensis* (IC_50_ = 60 mg/L)	[52]
		*Xylopia frutescens* Aubl.	6.8%	*Trypanosoma cruzi* (IC_50_ = 15 to 30 μg/mL)	[53]
	Cuba *	*Bursera graveolens* Triana & Planch	13%	*Leishmania amazonensis* (IC_50_ = 36.7 mg/L)	[54]
		*Piper auritum* Kunt	0.49%	*Leishmania major* (IC_50_ = 29.1 μg/mL) *Leishmania mexicana* (IC_50_ = 63.3 μg/mL) *Leishmania braziliensis* (IC_50_ = 52.1 μg/mL) *Leishmania donovani* (IC_50_ = 12.8 μg/mL)	[55]
(*Z*)-β-Ocimene	Brazil	*Syzygium cumini* (L.) Skeels.	29%	*Leishmania amazonensis* (60 mg/L)	[53]
	Cuba *	*Bursera graveolens* Triana & Planch	0.9%	*Leishmania amazonensis* (IC_50_ = 36.7 mg/L)	[54]
		*Piper ossanum* (C.DC.) Trel	0.14%	*Plasmodium falciparum* (IC_50_ = 1.5 µg/mL) *Trypanosoma brucei* (IC_50_ = 8.1 µg/mL) *Trypanosoma cruzi* (IC_50_ = 8.0 µg/mL) *Leishmania amazonensis* (IC_50_ = 19.3 µg/mL)	[47]
β-Phellandrene	Cameroon	*Ocimum gratissimum* L.	21.1%	*Plasmodium berghei* (at 200, 300 and 500 mg/kg caused a suppression of parasitaemia of 55.0%, 75.2% and 77.8%, respectively)	[56]
	Cuba *	*Bixa orellana* L.	0.2%	*Leishmania amazonensis* (8.5 mg/L)	[57]
		*Piper ossanum* (C.DC.) Trel	2.1%	*Plasmodium falciparum* (IC_50_ = 2.8 µg/mL) *Trypanosoma brucei* (IC_50_ = 8.4 µg/mL) *Trypanosoma cruzi* (IC_50_ = 8.6 µg/mL)	[47]
Camphor	Brazil	*Alpinia speciosa* K. Schum	17.1%	*Trypanosoma cruzi* (IC_50_ = 92 μg/mL) *Leishmania brasiliensis* (IC_50_ = 67 μg/mL)	[58]
		*Ocotea odorifera* (Vell) Rohwer	6.5%	*Leishmania amazonensis* (IC_50_ = 11.7 μg/mL)	[59]
		*Piper cubeba* L	5.6%	*Trypanosoma cruzi* (IC_50_ = 87.9 µg/mL)	[40]
	Cuba *	*Alpinia zerumbet* (Pers.) B.L.Burtt & R.M.Smith	0.1%	*Plasmodium falciparum* (IC_50_ = 66.2 µg/mL)	[45]
		*Piper aduncum* L.	17.1%	*Leishmania amazonensis* (IC_50_ = 23.8 μg/mL) *Leishmania donovani* (IC_50_ = 7.7 μg/mL) *Leishmania infantum* (IC_50_ = 8.1 μg/mL)	[46]
		*Piper ossanum* (C.DC.) Trel	13.8 and 9.4%	*Plasmodium falciparum* (IC_50_ = 1.5 and 2.8 µg/mL) *Trypanosoma brucei* (IC_50_ = 8.1 and 8.4 µg/mL) *Trypanosoma cruzi* (IC_50_ = 8.0 and 8.6 µg/mL)	[47]
	Ethiopia	*Artemisia absinthium* L.	27.4%	*Leishmania aethiopica* (IC_50_ = 8 μg/mL) *Leishmania donovani* (IC_50_ = 42 μg/mL)	[60]
	Morocco	*Rosmarinus officinalis* L.	18.7%	*Leishmania major* (IC_50_ = 1.2 μg/mL)	[61]
	Spain	*Artemisia absinthium* L.	4.5%	*Trypanosoma cruzi* (84% of inhibition at 200 μg/mL)	[62]
		*Artemisia pedemontana* subsp. *assoana* (Willk.) Rivas Mart.	7.7%	*Trypanosoma cruzi* (20 to 70% of inhibition at 200 μg/mL)	[62]
	Turkey	*Salvia recognita* Fisch. & Meyer	42%	*Plasmodium falciparum* (IC_50_ = 17 to 12 µg/mL)	[63]
	Tunisia	*Thymus hirtus* sp. *algeriensis* Boiss. et Reut	13.8%	*Leishmania major* (IC_50_ = 0.43 μg/mL) *Leishmania infantum* (IC_50_ = 0.25 μg/mL)	[39]
Germacrene D	Brazil	*Casearia sylvestris* Sw.	19.6%	*Leishmania amazonensis* (IC_50_ = 24.2 µg/mL)	[64]
		*Eugenia gracillima* Kiaersk.	16.1%	*Leishmania braziliensis* (IC_50_ = 74.6 µg/mL) *Leishmania infantum* (IC_50_ = 80.4 µg/mL)	[65]
		*Guatteria australis* A. St.-Hil.	22.2%	*Leishmania infantum* (IC_50_ = 30.7 μg/mL)	[66]
		*Lantana camara* L.	11.7%	*Trypanosoma cruzi* (IC_50_ = 201.94 μg/mL) *Leishmania braziliensis* (IC_50_ = 72.31 μg/mL)	[67]
		*Melampodium divaricatum* (Rich. ex Rich.) DC.	12.7%	*Leishmania amazonensis* (IC_50_ = 10.7 µg/mL)	[64]
		*Piper cernuum* Vell.	12.7%	*Leishmania amazonensis* (Infection index of 115 at 10 μg/mL)	[68]
		*Piper duckei* C. DC.	14.7%	*Leishmania amazonensis* (IC_50_ = 42–46 μg/mL) *Leishmania guyanensis* (IC_50_ = 15.2 μg/mL)	[69]
		*Vernonia polyanthes* (Spreng.) Vega & M. Dematteis	4.3%	*Leishmania infantum* (IC_50_ = 19.4 µg/mL)	[70]
		*Xylopia frutescens* Aubl.	17.8%	*Trypanosoma cruzi* (IC_50_ = 15 to 30 μg/mL)	[53]
	Cuba *	*Tagetes lucida* Cav.	0.3%	*Plasmodium berghei* (IC_50_ = 72 μg/mL)	[50]
Methyl eugenol	Brazil	*Aniba canelilla* (H.B.K.) Mez	14.8%	*Trypanosoma evansi* (Growth inhibition at 0.5%, 1.0% and 2.0%)	[71]
		*Hypenia salzmannii* (Benth.) Harley.	5.6%	*Trypanosoma cruzi* (IC_50_ = 35–42 µg/mL)	[72]
	Cuba *	*Piper auritum* Kunt	0.6%	*Leishmania major* (IC_50_ = 29.1 μg/mL) *Leishmania mexicana* (IC_50_ = 63.3 μg/mL) *Leishmania braziliensis* (IC_50_ = 52.1 μg/mL) *Leishmania donovani* (IC_50_ = 12.8 μg/mL)	[55]
	Bhutan	*Pleurospermum amabile* W.W.Smith,	3.8%	*Plasmodium falciparum* (IC_50_ = 79 μg/mL)	[73]
Piperitone	Benin	*Cymbopogon schoenantus* Spreng.	60.3%	*Trypanosoma brucei* (IC_50_ = 2.1 µg/mL) *Plasmodium falciparum* (IC_50_ = 43.1 µg/mL)	[49]
	Cuba *	*Alpinia zerumbet* (Pers.)B.L.Burtt&R.M.Smith	0.1%	*Plasmodium falciparum* (IC_50_ = 71.4 µg/mL)	[45]
		*Bursera graveolens* Triana & Planch	0.1%	*Leishmania amazonensis* (IC_50_ = 36.7 mg/L)	[54]
		*Piper aduncum* L.	23.7%	*Leishmania amazonensis* (IC_50_ = 23.8 μg/mL) *Leishmania donovani* (IC_50_ = 7.7 μg/mL) *Leishmania infantum* (IC_50_ = 8.1 μg/mL)	[46]
		*Piper ossanum* (C.DC.) Trel	20.1 and 19.0%	*Plasmodium falciparum* (IC_50_ = 1.5 and 2.8 µg/mL) *Trypanosoma brucei* (IC_50_ = 8.1 and 8.4 µg/mL) *Trypanosoma cruzi* (IC_50_ = 8.0 and 8.6 µg/mL)	[47]
Safrole	Brazil	*Myroxylon peruiferum* L.f.	8.3%	*Leishmania amazonensis* (IC_50_ = 54–162 μg/mL)	[74]
		*Ocotea odorifera* (Vell) Rohwer	6.5%	*Leishmania amazonensis* (IC_50_ = 11.7 μg/mL)	[59]
	Cuba *	*Piper auritum* Kunt	71.8%	*Leishmania major* (IC_50_ = 29.1 μg/mL) *Leishmania mexicana* (IC_50_ = 63.3 μg/mL) *Leishmania braziliensis* (IC_50_ = 52.1 μg/mL) *Leishmania donovani* (IC_50_ = 12.8 μg/mL)	[75]

* Data of EOs from Cuba used in this study.

## 3. Materials and Methods

### 3.1. Essential Oils Database

The 31 articles previously selected and analyzed by Monzote et al. [19] were used. A database with identified compounds with a concentration > 0.1% were performed and stored in Excel spreadsheet, which traces were not included. In parallel, the described pharmacological properties to each EO were assigned.

### 3.2. Machine Learning Analysis

#### 3.2.1. Self-Organizing Maps (SOMs)

The database contained information on 45 essential oils, with chemical composition and biological activity. For the realization of the neural maps, the information of the composition of the each EO from the dataset was used like descriptors. The chemical components were analyzed with SOMs in Matlab 6.5 and SOM Toolbox 2.0 [24]. The SOM Toolbox tool is a set of Matlab functions that can be used for the elaboration and implementation of neural networks, since it contains functions for the creation, visualization, and analysis of SOMs.

The data set was presented to the network before any adjustments were made. Subsequently, the data group was partitioned according to the regions of the weight vectors of the map, in each training stage. Then, the correct prediction of these sets and the total correct predictions of the compounds were evaluated. In the most relevant models, the set was divided into training and test sets to assess the forecasting capacity. Training and test performance were assessed by calculating the proportion of the number of samples correctly classified by SOM. For each map, 5 cross-validations were performed, being partitioned into 80% training and 20% testing. In the SOM, sites containing molecules for each descriptor were identified to highlight existing chemical patterns. The SOM was generated with a 4 × 6 rectangular GRID.

#### 3.2.2. Principal Component Analysis (PCA)

PCA analysis was calculated using the SOM toolbox 2.0 [24]. The utilization of PCA for dimension reduction lies in the fact that the PCs are generated so that they explain maximal amounts of variance [27].

The PCA was calculated using the database contained information on 45 essential oils.

#### 3.2.3. Random Forest Model

Knime 4.4.1 software (KNIME 4.4.1 the Konstanz Information Miner Copyright, 2021, www.knime.org, last accessed on 14 February 2022) [76,77] was used to perform the analyses and to generate the model, in silico. The EOs dataset were divided using a “Partitioning” tool, with the “Stratified sample” option, separated between training and testing datasets, which represented 80% and 20% of all compounds, respectively. Molecules in the training and testing datasets were randomly selected, but the same proportions of active and not reported substances were maintained for both databases. The information of the composition of the EOs was used like descriptors.

The model utilized a “5-fold external cross-validation” procedure and the Random Forest (RF) algorithm. The RF parameters selected for all models generated 100 total forests to be built, and −5,440,374,124,525,988,069 static random seeds (get reproducible results) were generated using random numbers for the model.

The external performances of the selected models were analyzed for sensitivity (true positive rate, which represents the active rate), specificity (true negative rate, which represents the inactive rate), and accuracy (general predictability).

The Applicability domain (APD) corresponds to the chemical space that surrounds the descriptors of the molecules used in the construction of the model. In this way, the applicability domain will provide information about the similarity between what is being tested and what was used to build the model [78,79,80].

The APD was used to assess whether predictions for the compounds in each dataset were reliable. The APD is based on Euclidean distances, and measures of similarity between the training set descriptors are used to define the APD. Therefore, if a compound in the test set has distances and similarities beyond the APD limit, its prediction will not be reliable. APD can be calculated using the following formula:

APD = d + Zσ(1)

where d and σ are the Euclidean distances for the mean and standard deviation of the compounds in the training set, respectively. Z is an empirical cutoff value, which was set to 0.5 in this study [81].

## 4. Conclusions

Scientific studies corroborate the results found in this study. Thus, this study of EO analysis establishes a way to find chemical pattern between EOs and antiparasitic activity (antileishmanial, antitrypanosomal and antimalarial). This finding makes it possible to direct studies and biological tests for EOs that have antiparasitic activity more effectively and with less time and financial cost. In particular, we strongly suggest further antiprotozoal studies with EOs from species of the *Piper* genus and the pure compound camphor taking into account data from Cuban EOs. Nevertheless, machine learning analysis studies will be interesting for EOs from different geographical locations to predict bioactive components with potential antiplasmodial, antileishmanial, and antitrypanosomal activity.

## Figures and Tables

**Table 1:** Accuracy statistics of the training and tests groups of the 5-fold external cross-validation of the Self-Organizing map (SOM).

**Classification of EOs**	**Training**					**Average**
	**1**	**2**	**3**	**4**	**5**	
True positive rate	0.90	0.95	0.99	0.90	0.80	0.91
True negative rate	0.70	0.65	0.68	0.70	0.75	0.71
Accuracy	0.81	0.80	0.83	0.81	0.78	0.81
**Classification of EOs**	**Test**					**Average**
	**1**	**2**	**3**	**4**	**5**	
True positive rate	0.60	0.60	0.95	0.60	0.99	0.75
True negative rate	0.75	0.75	0.70	0.95	0.80	0.80
Accuracy	0.67	0.67	0.83	0.77	0.90	0.78

**Table 2:** Summary of the statistics parameters of the RF model (average between the five models).

Model	Specificity	Sensitivity	Accuracy	PPV	NPV
RF	0.83	0.65	0.71	0.75	0.70

RF is random forest, PPV is positive predictive value, and NPV is negative predictive value.

**Table 3:** Summary of test averages corresponding to 5-fold cross-validation using the different machine learning algorithms, self-organizing maps (SOM) and random forest (RF).

Classification of EOs	Average	
	SOM (Unsupervised)	RF (Supervised)
Active	0.85	0.75
Not reported	0.83	0.70
Accuracy	0.84	0.71

## Data Availability

All data are available in the article and the supplementary material.

## Supplementary Materials

The following are available online, Table S1: Database of essential oils of Cuban plants.

## Sample Availability

There are no samples available.
