# Supplementary material for: Machine Learning Analysis of Essential Oils from Cuban Plants: Potential Activity against Protozoa Parasites
Source: Molecules. 2022 Feb 17;27(4):1366. doi: 10.3390/molecules27041366 (PMC8878085; doi:10.3390/molecules27041366)
Supplement: Supplementary file 1 [file molecules-27-01366-s001.zip › molecules-1536227-supplementary.pdf]

Table S1: Database of essential oils of Cuban plants.

No	Reference				Nat Prod Commun. 2014 Dec;9(12):1799-804.	ISRN Microbiol. 2012 Oct 14;2012:826786.	Pharmacologyonline 2(August 30):1-5 2015		Phytother Res. 2014 May;28(5):753-8.	Nat Prod Commun. 2012 Nov;7(11):1531	Journal of Essential Oil Research · October 2013	Nat Prod Commun. 2011 Feb;6(2):281			Chemotherapy. 2006;52(3):130-6.	Industrial Crops and Products 76 (2015) 647–652	ISRN Microbiol. 2012 Oct 14;2012:826786.	Environ Sci Pollut Res Int. 2020;27:9410-9423.	Nat Prod Commun. 2018 13(5):629-643	ISRN Microbiol. 2012 Oct 14;2012:826786.	Rev Bras Parasitol Vet. 2015 Jan-Mar;24(1):36-44.	Chem Biodivers. 2016 Mar;13(3):336-342.	ISRN Microbiol. 2012 Oct 14;2012:826786.	Journal of Essential Oil Research 2014, 26(4)	Molecules. 2020;25:5514.
	Plant				Artemisia absinthium	Allium sativum	Alpinia zerumbet		Bixa orellana	Bursera graveolens	Callistemon speciosus	Chenopodium ambrosioides			Chenopodium ambrosioides	Citrus sinensis	Citrus sinensis	Croton linearis	Croton linearis	Cuminum cyminum	Cymbopogon citratus	Lantana camara	Laurus nobilis	Licaria triandra	Melaleuca leucadendra
							leaves	flowers				C1 (green material)	C2 (dry at room temperature)	C3 (fermented in water for 3 days)											
Biological activity	NONE																			x			x		
	Antibacterial					x					x						x					x		x	
	Anticancer									x															x
	Antifungal					x																			
	Anthelminthic															x									
	Antileishmanial				x				x	x		x	x	x	x				x						x
	Antioxidant																								
	Antiplasmodial						x	x																	
	Antitrypanosomal																								
	Insecticide																								
	Larvicidal effect																	x			x				
	Oxidative stress																								
	Compound	MF	MM	chemical clasification	%	%	%	%	%	%	%	%	%	%	%	%	%	%	%	%	%	%	%	%	%
1	methyl 2-propenyl sulfide	C4H8S	88	sulfur compound		5.6																			
2	(E)-2-hexanal	C6H10O	98	aliphatic																					
3	(2Z)-Hexenol	C6H12O	100	aliphatic						0.1															
4	(3E)-Hexenol	C6H12O	100	aliphatic								0.1													
5	(3Z)-Hexen-1-ol =[(3Z)-Hexenol]	C6H12O	100	aliphatic						0.5		0.1		0.1										0.1	0.1
6	Hexanal	C6H12O	100	aliphatic																				0.1	
7	1-Hexanol	C6H14O	102	aliphatic								0.1		0.3										0.1	
8	Benzaldehyde	C7H6O	106	phenylpropanoids																				0.1	0.1
9	p-Cresol	C7H8O	108	phenylpropanoids										0.2											
10	di-2-propenyl sulfide	C6H10S	114	sulfur compound		7.9																			
11	2-heptanol	C7H16O	116	aliphatic				0.1																	
12	Indole	C8H7N	117	alkaloid																					
13	methyl 2-propenyl disulfide	C4H8S2	120	sulfur compound		5.6																			
14	(E)-6-Methyl-3,5-heptadien-2-one	C8H12O	124	aliphatic						0.1															
15	6-methylhept-5-en-2-one =[Sulcatone]	C8H14O	126	aliphatic																	0.23				
16	dimethyl trisulfide	C2H6S3	126	sulfur compound		1.6																			
17	1-Octen-3-ol	C8H16O	128	aliphatic																		0.5			
18	Octan-3-ol	C8H18O	130	aliphatic																		0.2			
19	octanol	C8H18O	130	aliphatic																					
20	p-Cymenene	C10H12	132	monoterpene								0.2	0.1	0.1											
21	1,3,8-p-Menthatriene	C10H14	134	monoterpene								0.1		0.1											
22	p-Cymene	C10H14	134	monoterpene	2.6			0.1			3.1	21.3	20.2	21.1	0.83			3.37		2.7		1.3		0.4	0.7
23	(+)-sabinene	C10H16	136	monoterpene																					
24	Methyl benzoate	C8H8O2	136	phenylpropanoids							0.1														0.1
25	Carbicol	C9H12O	136	monoterpene																4.4					
26	(E)-β-Ocimene	C10H16	136	monoterpene						13								0.1				0.2		0.1	
27	(Z)-β-Ocimene	C10H16	136	monoterpene						0.9								0.28						0.1	
28	Camphene	C10H16	136	monoterpene																		1.5		2.4	0.1
29	D-limonene	C10H16	136	monoterpene													82.7								
30	Limonene	C10H16	136	monoterpene						26.5	4.8	0.6	0.5	0.6		96		0.91				1.3		2.9	4.8
31	Myrcene =[β-Myrcene]	C10H16	136	monoterpene				0.1	0.1	0.7	0.2		0.1	0.1				1.32			6.52	0.5		2.2	0.5
32	Sabinene	C10H16	136	monoterpene				1.9		0.3								9.37	0.21			2	11.8		
33	Sylvestrene	C10H16	136	monoterpene																					
34	terpinene	C10H16	136	monoterpene				0.8																	
35	Terpinolene	C10H16	136	monoterpene				0.2			0.1							0.28						0.5	0.1
36	Thuja-2,4(10)-diene	C10H16	136	monoterpene																				0.1	
37	Tricyclene	C10H16	136	monoterpene																				0.3	
38	α-3-Carene	C10H16	136	monoterpene																			0.1		
39	α-ocimene	C10H16	136	monoterpene																					
40	α-Phellandrene	C10H16	136	monoterpene						0.1	1.2							0.44				0.1		0.1	0.1
41	α-Pinene	C10H16	136	monoterpene					3.3	0.1	7.7					0.14	2.4	1.52				3.2		14.8	2.7
42	α-Terpinene	C10H16	136	monoterpene				0.3				20.7	19.7	17				1.44		4.5				0.4	
43	α-Thujene	C10H16	136	monoterpene							0.2							1.09				0.1			
44	β-ocimene	C10H16	136	monoterpene																					
45	β-Phellandrene	C10H16	136	monoterpene					0.2													0.3			
46	β-Pinene	C10H16	136	monoterpene					2.3	0.2	0.5							0.76		12.8		2.5	2.4	18.2	1.2
47	γ-Terpinene	C10H16	136	monoterpene							0.2	0.5	0.4	0.5				1.18	0.13	2.5		0.1		0.7	0.2
48	δ-3-Carene	C10H16	136	monoterpene										0.1										0.2	
49	Δ^4-carene 3,7,7-trimethylbicycle	C10H16	136	monoterpene											0.76										
50	Cryptone	C9H14O	138	monoterpene																					
51	di-2-propenyl disulfide	C6H10S2	146	sulfur compound		20.7																			
52	(E)-Anethole =[Anethole]	C10H12O	148	monoterpene																					
53	3-isopropylbenzaldehyde	C10H12O	148	monoterpene																					
54	Coahuilensol methyl ether	C10H12O	148	monoterpene																					
55	Cuminaldehyde =[cuminal]	C10H12O	148	monoterpene				0.4												63.7					
56	Estragole =[chavicol o methyl chavicol]	C10H12O	148	monoterpene																					
57	acetanisole	C9H10O2	150	monoterpene																					
58	Carvacrol	C10H14O	150	monoterpene	3.2		0.2					4.2	0.1	1.4	62.36										
59	Carvone	C10H14O	150	monoterpene						0.1															
60	Menthofuran	C10H14O	150	monoterpene						5.1															
61	Myrtenal	C10H14O	150	monoterpene																					
62	p-Cymen-7-ol	C10H14O	150	monoterpene	5.4																				
63	p-Cymen-8-ol	C10H14O	150	monoterpene	1.0							0.6	0.2	0.4											
64	p-Cymen-9-ol	C10H14O	150	monoterpene								0.5	0.2												
65	Pinocarvone	C10H14O	150	monoterpene																				0.2	
66	Piperitenone	C10H14O	150	monoterpene						0.1															
67	Thymol	C10H14O	150	monoterpene								0.2	0.2	2.4					0.05	2.3					
68	cis/trans-Isopulegone	C10H16O	152	monoterpene						0.7															
69	(-)-Camphor	C10H16O	152	monoterpene																					
70	methyl o-hydroxibenzoate	C8H8O3	152	phenylpropanoids																					
71	(E)-Epoxyocimene	C10H16O	152	monoterpene						0.4															
72	(Z)-Chrysanthenole	C10H16O	152	monoterpene				0.1																	
73	(Z)-Epoxyocimene	C10H16O	152	monoterpene						0.2															
74	Camphor	C10H16O	152	monoterpene				0.1																	
75	Carvenone	C10H16O	152	monoterpene			0.1																		
76	cis-Carveol	C10H16O	152	monoterpene						0.1															
77	cis-Pinocarveol	C10H16O	152	monoterpene																					
78	citral	C10H16O	152	monoterpene													1.8								
79	E-citral (geranial)	C10H16O	152	monoterpene																51.14					
80	Fenchone	C10H16O	152	monoterpene																					
81	Limonen-10-ol	C10H16O	152	monoterpene						0.1															
82	Myrtenol	C10H16O	152	monoterpene			0.7																	0.4	
83	Perilla alcohol	C10H16O	152	monoterpene						0.1															
84	Piperitone	C10H16O	152	monoterpene			0.1			0.1												0.6			
85	p-mentha-1,4-dien-7-ol	C10H16O	152	monoterpene			0.3	0.1																	
86	Pulegone	C10H16O	152	monoterpene						1.7															
87	trans-Carveol	C10H16O	152	monoterpene						0.3															
88	trans-Dihydrocarvone	C10H16O	152	monoterpene						0.1															
89	trans-Pinocarveol	C10H16O	152	monoterpene																				0.4	
90	trans-p-Mentha-2,8-dien-1-ol	C10H16O	152	monoterpene						0.1		0.5	0.2	0.3											
91	trans-Sabinol	C10H16O	152	monoterpene	5.0														0.1						
92	trans-Verbenol	C10H16O	152	monoterpene																		0.1		0.2	
93	Z-citral (Neral)	C10H16O	152	monoterpene																	35.21				
94	α-Pinene oxide	C10H16O	152	monoterpene						0.4															
95	methyl 2-propenyl trisulfide	C4H8S3	152	sulfur compound		21.7																			
96	trans-pinene hydrate	C10H18O	154	monoterpene																					
97	δ-Terpineol	C10H18O	154	monoterpene							16.6							0.35				0.1			15.6
98	4-Hydroxycryptone	C9H14O2	154	monoterpene																					
99	(Z)-2-p-Menthen-1-ol	C10H18O	154	monoterpene																				0.1	
100	1,8-Cineole (eucalyptol)	C10H18O	154	monoterpene				5			6.1							26.66	2.45			6.9	26.7	0.4	61
101	4-Thujanol	C10H18O	154	monoterpene														0.19							
102	Borneol	C10H18O	154	monoterpene	4.8			0.1			0.1											0.3		0.3	0.1
103	Camphene hydrate	C10H18O	154	monoterpene																				0.1	
104	cis-sabinene hydrate	C10H18O	154	monoterpene			0.5	1.1																0.2	
105	cis-β-terpineol	C10H18O	154	monoterpene																					
106	Citronellal	C10H18O	154	monoterpene																	0.16				
107	endo-Fenchol	C10H18O	154	monoterpene							0.1													0.1	0.1
108	Geraniol	C10H18O	154	monoterpene			0.1	0.1					0.3	0.7							2.23				
109	Isoborneol	C10H18O	154	monoterpene																		0.5			
110	Lavandulol	C10H18O	154	monoterpene																					
111	Linalool	C10H18O	154	monoterpene				3.2			0.2			0.1				2.22	0.04		0.69	0.9	18.5	0.5	0.2
112	Menthone	C10H18O	154	monoterpene						0.3															
113	neo-Dihydrocarveol	C10H18O	154	monoterpene						0.1															
114	neo-Isopulegol	C10H18O	154	monoterpene							0.4														0.4
115	Nerol	C10H18O	154	monoterpene																					
116	Terpinen-1-ol	C10H18O	154	monoterpene								0.7	0.1	0.6											
117	trans-2-p-menthen-1-ol	C10H18O	154	monoterpene			1.6	0.6				0.2	---	0.6											
118	trans-Dihydro rose oxide	C10H18O	154	monoterpene								0.2	0.1	0.1											
119	trans-Piperitol	C10H18O	154	monoterpene			1.5	0.4																	
120	trans-sabinene hydrate	C10H18O	154	monoterpene			4.1																		
121	Yomogi alcohol	C10H18O	154	monoterpene																					
122	α-Terpineol	C10H18O	154	monoterpene	2.4		4.3	3.6		0.3	0.3			2.4				4.38	1.11			1.7	1.2	1.1	0.3
123	cis-piperitol	C10H18O	154	monoterpene				0.2																	
124	cis-p-Menth-2-en-1-ol	C10H18O	154	monoterpene			1.3	0.7										0.12							
125	iso-Isopulegol	C10H18O	154	monoterpene							0.1														0.1
126	Terpinen-4-ol	C10H18O	155	monoterpene	0.2		19	14.1			1.2	0.3		0.3				1.55	0.52			0.5		1.2	1.2
127	2-Decanone	C10H20O	156	monoterpene																				0.1	
128	Citronellol =[β-Citronellol]	C10H20O	156	monoterpene				0.2			0.1						1.5								0.1
129	(E)-p-Methoxycinnamaldehyde	C10H10O2	162	monoterpene																					
130	methyl (E)-cinnamate	C10H10O2	162	monoterpene				0.1																	
131	Safrole	C10H10O2	162	monoterpene																					
132	Eugenol	C10H12O2	164	phenylpropanoids														0.11					18.5		
133	Thymol methyl ether	C11H16O	164	monoterpene														0.41	0.13						
134	Dihydroisojasmone	C11H18O	166	monoterpene										0.6											
135	trans-Piperitone epoxide	C10H16O2	168	monoterpene								1	0.7	1.4											
136	Ascaridole	C10H16O2	168	monoterpene								35.1	47.1	30.5	22.54										
137	cis-Piperitone epoxide	C10H16O2	168	monoterpene								0.8	0.4	0.8											
138	Isoascaridol	C10H16O2	168	monoterpene								0.5	5	1.5											
139	Isoascaridole	C10H16O2	168	monoterpene											1.86										
140	cis-Linalool oxide (furanoid)	C10H18O2	170	monoterpene																					
141	trans-Ascaridol glycol	C10H18O2	170	monoterpene	2.0							0.2	1	4.5											
142	trans-Linalool oxide (furanoid)	C10H18O2	170	monoterpene																					

No	Reference	Chem Biodivers. 2010 Sep;7(9):2281-8.		Industrial Crops and Products 76 (2015) 647–652	Nat Prod Commun. 2012 Nov;7(11):1527-30.	Journal of Essential Oil Research 2014, 26:5, 359-362,	International Journal of Pharmacognosy and Phytochemical Research 2017; 9(7); 1021-1028	ISRN Microbiol. 2012 Oct 14;2012:826786.	Molecules. 2019 Apr 24;24(8):1615.	Rev Cubana Med Trop. 2007 May-Aug;59(2):154-8.	ISRN Microbiol. 2012 Oct 14;2012:826786.	Medicines (Basel). 2017 Jul 2;4(3):49.	Nat Prod Commun. 2013 Sep;8(9):1325-8.	Chem Biodivers. 2016 Dec;13(12):1715-1719.		Mem Inst Oswaldo Cruz. 2010 Mar;105(2):168-73.	Nat Prod Commun. 2013 Sep;8(9):1325-8.	Nat Prod Commun. 2013 Sep;8(9):1325-8.	Nat Prod Communic 2020	Phytother Res. 2017 Sep;31(9):1419	ISRN Microbiol. 2012 Oct 14;2012:826786.	Journal of Essential Oil Research 2011, 23:5, 63-67	Sci. Pharm. 2020;88(31):1-8	Pharmacogn Mag. 2010 Jul;6(23):242-7.	Current Topics in Phytochemistry 2020;16
	Plant	Melaleuca leucadendra		Melaleuca quinquenervia	Murraya paniculata	Nectandra antillana	Ocimum sanctum	Origanum vulgare	Phania matricarioides	Pimenta racemosa	Pimpinella anisum L.	Piper aduncum	Piper aduncum	Piper aduncum var. ossanum		Piper auritum	Piper auritum	Piper umbellatum	Plectranthus amboinicus	Pluchea carolinensis	Syzygium aromaticum L.	Tagetes lucida	Tagetes lucida	Tamarindus indica	Zanthoxylum pseudodumosum
		leaf	fruit											from Bauta (EO-B)	from Ceiba (EO-C)										
Biological activity	NONE						x																		
	Antibacterial				x	x		x						x							x	x		x	
	Anticancer																		x						
	Antifungal							x			x													x	x
	Anthelminthic			x																					
	Antileishmanial				x				x			x		x	x	x				x			x		
	Antioxidant	x	x										x				x	x							
	Antiplasmodial								x			x		x	x				x			x			
	Antitrypanosomal								x			x		x	x										
	Insecticide									x															
	Larvicidal effect																								
	Oxidative stress																								
	Compound		%	%	%	%	%	%	%	%	%	%	%	%	%	%	%	%	%	%	%	%	%	%	%
1	methyl 2-propenyl sulfide																								
2	(E)-2-hexanal																							1.7	
3	(2Z)-Hexenol																								
4	(3E)-Hexenol																								
5	(3Z)-Hexen-1-ol =[(3Z)-Hexenol]																								
6	Hexanal																								
7	1-Hexanol																								
8	Benzaldehyde	0.3	0.3				0.44																		
9	p-Cresol																								
10	di-2-propenyl sulfide																								
11	2-heptanol																								
12	Indole				3.5																				
13	methyl 2-propenyl disulfide																								
14	(E)-6-Methyl-3,5-heptadien-2-one																								
15	6-methylhept-5-en-2-one =[Sulcatone]																								
16	dimethyl trisulfide																								
17	1-Octen-3-ol									1.5									0.6	0.1					
18	Octan-3-ol																								
19	octanol									0.3															
20	p-Cymenene									0.2															
21	1,3,8-p-Menthatriene																								
22	p-Cymene		0.1			0.4		3.7		8		1		0.61		2.2	0.23		9.7	0.1				0.4	
23	(+)-sabinene																0.08								
24	Methyl benzoate																								
25	Carbicol																								
26	(E)-β-Ocimene					0.1								0.28								0.2 ± 0.01	0.1	t	
27	(Z)-β-Ocimene													0.14											
28	Camphene	0.2	0.1			0.9						5.9	10.9	7.41	5.39	5.5		2.1							0.44
29	D-limonene																								
30	Limonene	4.8	3.9			3.5						1.5					0.75	0.9						24.7	
31	Myrcene =[β-Myrcene]	0.2				0.4				1.2		0.1		0.5	0.46		0.28		0.6			2.3 ± 0.04	1.1		
32	Sabinene							3.7		0.3								2.4		0.1					
33	Sylvestrene													2.49											
34	terpinene																								
35	Terpinolene	0.2	0.1			0.7				3.1						0.6	0.37								
36	Thuja-2,4(10)-diene																								
37	Tricyclene																								
38	α-3-Carene																								
39	α-ocimene																0.09								
40	α-Phellandrene					1				1.6				0.12	0.65										
41	α-Pinene	5.3	4.5			0.5				2		0.8	2.9	1.67	1.36	3.8	86.91	3.6		6.1				0.9	
42	α-Terpinene							4.3		0.1							0.08		0.6						
43	α-Thujene									1.1									0.8						
44	β-ocimene																0.49								
45	β-Phellandrene														2.12										
46	β-Pinene	2.7	0.9			0.4				0.3		0.4		0.49	0.26	1.4		1.4						1.3	
47	γ-Terpinene	0.3	0.1			0.1		7.3		4.6						0.7	0.49		4.3						
48	δ-3-Carene					0.1												0.2							
49	Δ^4-carene 3,7,7-trimethylbicycle																								
50	Cryptone											0.5													
51	di-2-propenyl disulfide									0.1	80.8										5.9		0.1		
52	(E)-Anethole =[Anethole]								0.2																
53	3-isopropylbenzaldehyde								0.7																
54	Coahuilensol methyl ether																								
55	Cuminaldehyde =[cuminal]											0.1													
56	Estragole =[chavicol o methyl chavicol]									10.18													0.1		
57	acetanisole										2														
58	Carvacrol							3.4											71						
59	Carvone											0.1													
60	Menthofuran																								
61	Myrtenal											0.1													
62	p-Cymen-7-ol																								
63	p-Cymen-8-ol																								
64	p-Cymen-9-ol																								
65	Pinocarvone											0.1													
66	Piperitenone																								
67	Thymol							38	1.2			0.1				0.6									
68	cis/trans-Isopulegone																								
69	(-)-Camphor												17.1					9.6							
70	methyl o-hydroxibenzoate																				9				
71	(E)-Epoxyocimene																								
72	(Z)-Chrysanthenole								0.2																
73	(Z)-Epoxyocimene																								
74	Camphor					1.2						17.1		13.87	9.41										
75	Carvenone																								
76	cis-Carveol																								
77	cis-Pinocarveol																								
78	citral																								
79	E-citral (geranial)									0.2															
80	Fenchone											0.1													
81	Limonen-10-ol																								
82	Myrtenol																			0.1					
83	Perilla alcohol																								
84	Piperitone											23.7	34	20.07	19.01										
85	p-mentha-1,4-dien-7-ol																								
86	Pulegone																								
87	trans-Carveol	0.1										0.1													
88	trans-Dihydrocarvone																								
89	trans-Pinocarveol	0.2	0.1						0.2			0.1													
90	trans-p-Mentha-2,8-dien-1-ol																								
91	trans-Sabinol																								
92	trans-Verbenol																			0.1					
93	Z-citral (Neral)																								
94	α-Pinene oxide																								
95	methyl 2-propenyl trisulfide																								
96	trans-pinene hydrate																	1.7							
97	δ-Terpineol																								
98	4-Hydroxycryptone											0.1													
99	(Z)-2-p-Menthen-1-ol																								
100	1,8-Cineole (eucalyptol)	43	3.6	25.43			0.13			20.4		0.1	8.7					3.6							
101	4-Thujanol																								
102	Borneol	0.4	0.1									0.4		0.85	0.6										
103	Camphene hydrate	0.1										0.5													
104	cis-sabinene hydrate																								
105	cis-β-terpineol							16.5																	
106	Citronellal																								
107	endo-Fenchol	0.2	0.1																						
108	Geraniol																								
109	Isoborneol											3.6	4.3	3.94	3.91			2.8							
110	Lavandulol								5.5																
111	Linalool	0.4					7.13		0.3	1.3	2.3	1.2	1.3	0.72	0.49	0.9	0.68	3.6		0.7		0.1 ± 0.01	0.2	1.1	
112	Menthone																								
113	neo-Dihydrocarveol																								
114	neo-Isopulegol	0.2																							
115	Nerol																							1.5	
116	Terpinen-1-ol																								
117	trans-2-p-menthen-1-ol																								
118	trans-Dihydro rose oxide																								
119	trans-Piperitol											0.1													
120	trans-sabinene hydrate																								
121	Yomogi alcohol								0.1																
122	α-Terpineol	7	0.9							10		0.7	1.6	1.17	0.71	0.2		1.1						0.5	
123	cis-piperitol																								
124	cis-p-Menth-2-en-1-ol																								
125	iso-Isopulegol																								
126	Terpinen-4-ol	1.1	0.2							20.7		0.2		0.18				0.9	1.4	0.1					
127	2-Decanone								0.1																
128	Citronellol =[β-Citronellol]																								
129	(E)-p-Methoxycinnamaldehyde																						0.4		
130	methyl (E)-cinnamate																								
131	Safrole															71.8	1.59	26.4							
132	Eugenol					0.2	21.96			10.7							0.05				67				
133	Thymol methyl ether					0.1														0.1					
134	Dihydroisojasmone																								
135	trans-Piperitone epoxide																								
136	Ascaridole																								
137	cis-Piperitone epoxide																								
138	Isoascaridol																								
139	Isoascaridole																								
140	cis-Linalool oxide (furanoid)											0.1													
141	trans-Ascaridol glycol																								
142	trans-Linalool oxide (furanoid)											0.1													

143	2-undecanone	$C_{11}H_{22}O$	170	aliphatic																0.35																													
144	2-Methylbutyl 2-methylbutanoate	$C_{10}H_{20}O_2$	172	monoterpene																		0.1																											
145	Herniarin	$C_{10}H_8O_3$	176	monoterpene																																											0.1		
146	(E)-Methyl isoeugenol	$C_{11}H_{14}O_2$	178	monoterpene																				0.6																									
147	(Z)-Methyl isoeugenol	$C_{11}H_{14}O_2$	178	monoterpene																				0.1																									
148	Methyl eugenol	$C_{11}H_{14}O_2$	178	monoterpene																			6.5							3.6			0.3		2.4					0.6		0.6							
149	Di-2-propenyl trisulfide	$C_6H_{10}S_3$	178	sulfur compound		31.9																																											
150	Z3,Z6,E8-dodecatrien-1-ol	$C_{12}H_{20}O$	180	aliphatic																																					0.74								
151	Bornylformate	$C_{11}H_{18}O_2$	182	monoterpene																																0.1													
152	Isobornylformate	$C_{11}H_{18}O_2$	182	monoterpene																																0.6													
153	Methyl 2,4-decadienoate	$C_{11}H_{18}O_2$	182	aliphatic																																					0.33								
154	Hexyl tiglate	$C_{11}H_{20}O_2$	184	monoterpene											1.01																																		
155	4-Methyl-5-thiazole ethanol acetate	$C_8H_{11}NO_2S$	185	aliphatic	0.7																																												
156	Methyl decanoate	$C_{11}H_{22}O_2$	186	monoterpene																																					0.53								
157	Myristicine	$C_{11}H_{12}O_3$	192	monoterpene																																					0.11								
158	4-phenyl-2-butyl acetate =[Benzenepropanol, α-methyl-, acetate]	$C_{12}H_{16}O_2$	192	monoterpene			0.1																																										
159	Carvacryl acetate	$C_{12}H_{16}O_2$	192	monoterpene																																							0.1						
160	(E)-β-Ionone	$C_{13}H_{20}O$	192	monoterpene										0.4																																			
161	2,5-Dimethoxy-p-cymene	$C_{12}H_{18}O_2$	194	monoterpene						0.1																																		7.5					
162	4-allylsyringol	$C_{11}H_{14}O$	194	monoterpene																													0.3																
163	Thymohydroquinone dimethyl ether	$C_{12}H_{18}O_2$	194	monoterpene																													1.2																
164	trans-Sabinyl acetate	$C_{12}H_{18}O_2$	194	monoterpene	36.7																									0.3																			
165	trans-Verbenyl acetate	$C_{12}H_{18}O_2$	194	monoterpene														0.05																															
166	Bornyl acetate	$C_{12}H_{20}O_2$	196	monoterpene			0.5	0.5										0.05						0.7						0.3						0.1													
167	Geranyl acetate	$C_{12}H_{20}O_2$	196	monoterpene				0.1												0.2																													
168	Lavandulyl acetate	$C_{12}H_{20}O_2$	196	monoterpene																													40.1																
169	Neryl acetate	$C_{12}H_{20}O_2$	196	monoterpene																													0.2																
170	α-Terpinyl acetate	$C_{12}H_{20}O_2$	196	monoterpene				1.4														0.1				1.6	0.3																						
171	β-Terpinyl acetate	$C_{12}H_{20}O_2$	196	monoterpene														0.09																															
172	Neomentil acetate	$C_{12}H_{22}O_2$	198	aliphatic											0.62																																		
173	2- tridecanone	$C_{13}H_{26}O$	198	aliphatic																0.1																													
174	Tridecanal	$C_{13}H_{26}O$	198	aliphatic																																								0.6					
175	Tetradecane	$C_{14}H_{30}$	198	aliphatic																																					0.13								
176	α-Calacorene	$C_{15}H_{20}$	200	sesquiterpene															0.85			0.1		0.1						0.3						0.4													
177	β-Calacorene	$C_{15}H_{20}$	200	sesquiterpene															1.04											1.2																			
178	ar-Curcumene	$C_{15}H_{22}$	202	sesquiterpene																																0.4													
179	Silphiperfol-4,7(14)-diene	$C_{15}H_{22}$	202	sesquiterpene																													0.4																
180	trans-Calamenene	$C_{15}H_{22}$	202	sesquiterpene			1.3	0.5														0.1																											
181	Selina-4,11-diene (= γ-Selinene)	$C_{15}H_{24}$	204	sesquiterpene																																								0.3					
182	(E)-Caryophyllene	$C_{15}H_{24}$	204	sesquiterpene														0.64																										2					32.01
183	(E)-β-Farnesene	$C_{15}H_{24}$	204	sesquiterpene						0.1												11.3																									0.1		
184	(E)-γ-Bisabolene	$C_{15}H_{24}$	204	sesquiterpene														0.16																															
185	(E,E)-α-Farnesene	$C_{15}H_{24}$	204	sesquiterpene																																										0.1 ± 0.01	0.1		
186	(Z)-β-Farnesene	$C_{15}H_{24}$	204	sesquiterpene																		0.1																											
187	2-epi-(E)-β-Caryophyllene	$C_{15}H_{24}$	204	sesquiterpene															0.42																														
188	4,5-di-epi-Aristolochene	$C_{15}H_{24}$	204	sesquiterpene						0.2																																							
189	7-epi-Sesquithujene	$C_{15}H_{24}$	204	sesquiterpene														0.11																															
190	7-epi-silphiperfol-5-ene	$C_{15}H_{24}$	204	sesquiterpene																													1.8																
191	7-epi-α-Selinene	$C_{15}H_{24}$	204	sesquiterpene					0.9	0.1									2.93																														
192	9-epi-(E)-Caryophyllene	$C_{15}H_{24}$	204	sesquiterpene														0.26												0.1																			
193	Alloaromadendrene	$C_{15}H_{24}$	204	sesquiterpene				0.3											0.5			0.6					0.3	9.5			1.16		0.3																3.16
194	Aristolene	$C_{15}H_{24}$	204	sesquiterpene					0.9																																								
195	Aristolochene	$C_{15}H_{24}$	204	sesquiterpene					1																																			1.4					
196	Aromadendrene	$C_{15}H_{24}$	204	sesquiterpene					0.2																		0.1			0.2						0.8		0.56	0.31		0.09								
197	Bicyclogermacrene	$C_{15}H_{24}$	204	sesquiterpene					8.4															0.6					5.6	1.4	20.38		3.7					1.24	1.66			0.5							
198	Cadina-1,4-diene	$C_{15}H_{24}$	204	sesquiterpene															3.48																														
199	cis-β-Guaiene	$C_{15}H_{24}$	204	sesquiterpene																							0.3																						
200	cis-Cadina-1(6),4-diene	$C_{15}H_{24}$	204	sesquiterpene						0.1																																							
201	cis-Muurola-4(14),5-diene	$C_{15}H_{24}$	204	sesquiterpene						0.2																				0.1																			
202	Cycloisosativene	$C_{15}H_{24}$	204	sesquiterpene														0.17																															
203	Cyclosativene	$C_{15}H_{24}$	204	sesquiterpene																																0.1													
204	daucene	$C_{15}H_{24}$	204	sesquiterpene			0.1	0.2																																									
205	Eremophilene	$C_{15}H_{24}$	204	sesquiterpene														0.34																															
206	Germacrene	$C_{15}H_{24}$	204	sesquiterpene																																					0.3					0.3 ± 0.02			
207	Germacrene A	$C_{15}H_{24}$	204	sesquiterpene					0.2	3.9																																							
208	Guaia-6,9-diene	$C_{15}H_{24}$	204	sesquiterpene																		0.1																											
209	Ishwarane	$C_{15}H_{24}$	204	sesquiterpene					18.6																																								
210	Isoledene	$C_{15}H_{24}$	204	sesquiterpene						0.1									0.02																														
211	Longicyclene	$C_{15}H_{24}$	204	sesquiterpene															0.47																														
212	Longifolene	$C_{15}H_{24}$	204	sesquiterpene														0.41									0.1	32.95																					
213	Modheph-2-ene	$C_{15}H_{24}$	204	sesquiterpene																																								0.1					
214	Myltayl-4(12)-ene	$C_{15}H_{24}$	204	sesquiterpene					0.9																																								
215	Selina-3,7(11)-diene	$C_{15}H_{24}$	204	sesquiterpene														0.72																															
216	Selina-4(15),7(11)-diene	$C_{15}H_{24}$	204	sesquiterpene															4.19																														
217	Silphiperfol-5-ene	$C_{15}H_{24}$	204	sesquiterpene																													0.3																
218	trans-Cadina-1(2),4-diene	$C_{15}H_{24}$	204	sesquiterpene																				0.2						0.3																			
219	trans-Cadina-1(6),4-diene	$C_{15}H_{24}$	204	sesquiterpene																		0.4		0.1																									
220	trans-Cadina-1,4-diene	$C_{15}H_{24}$	204	sesquiterpene						0.1																																							
221	Trans-caryophyllene	$C_{15}H_{24}$	204	sesquiterpene																																					0.07								
222	trans-Muurola-3,5-diene	$C_{15}H_{24}$	204	sesquiterpene																				0.1																									
223	trans-Muurola-4(14),5-diene	$C_{15}H_{24}$	204	sesquiterpene																		0.1																											
224	trans-α-Bergamotene	$C_{15}H_{24}$	204	sesquiterpene			0.3	0.1																																			2.7						
225	trans-β-Bergamotene	$C_{15}H_{24}$	204	sesquiterpene																																											0.1		
226	trans-β-Guaiene	$C_{15}H_{24}$	204	sesquiterpene					0.2	1.8																																							
227	Valencene	$C_{15}H_{24}$	204	sesquiterpene																																								0.8					
228	Viridiflorene	$C_{15}H_{24}$	204	sesquiterpene																						0.3	2.9			0.3						0.4													
229	Zonarene	$C_{15}H_{24}$	204	sesquiterpene														1.04						0.1																									
230	α-Amorphene	$C_{15}H_{24}$	204	sesquiterpene														0.74	3.68																														
231	α-Bulnesene	$C_{15}H_{24}$	204	sesquiterpene																										0.5																			
232	α-Cadinene	$C_{15}H_{24}$	204	sesquiterpene				0.1	0.3										0.09					0.2			0.1			0.1						0.1													
233	α-Caryophyllene =[α-Humulene]	$C_{15}H_{24}$	204	sesquiterpene			1.8	0.5	0.4	0.3								0.44	1.49					1.1			0.3		5.3	4.1	4.2		1.1					2.24	1.37		0.13		1.3	0.1					
234	α-Copaene	$C_{15}H_{24}$	204	sesquiterpene					1.2	2.3												1.5		0.4			0.1		3.8	4.7			3.2			0.5		0.25	0.13	0.9	0.55	0.6							
235	α-Cubebene	$C_{15}H_{24}$	204	sesquiterpene					0.1															0.1					2.2	0.2																			0.46
236	α-Guaiene	$C_{15}H_{24}$	204	sesquiterpene			0.1	0.1		0.1								0.14				0.2								0.2																			1.14
237	α-Gurjunene	$C_{15}H_{24}$	204	sesquiterpene						0.2																												0.48	0.21										
238	α-muurolene	$C_{15}H_{24}$	204	sesquiterpene				0.3											1.43			0.7		0.2			0.2			0.8						0.5													
239	α-Santalene	$C_{15}H_{24}$	204	sesquiterpene																																0.2													
240	α-Selinene	$C_{15}H_{24}$	204	sesquiterpene				0.5		2.4								0.22				1.7																											8.67
241	α-trans Bergamotene	$C_{15}H_{24}$	204	sesquiterpene																											0.39																		
242	α-Ylangene	$C_{15}H_{24}$	204	sesquiterpene					0.2															0.1						0.5						0.1													
243	β- Bisabolene	$C_{15}H_{24}$	204	sesquiterpene				0.1														0.7									4.12																		
244	Germacrene B	$C_{15}H_{24}$	204	sesquiterpene					0.2									0.34				0.5		1.4						0.6	0.26							0.74	0.81										
245	β-Acoradiene	$C_{15}H_{24}$	204	sesquiterpene															0.25																														
246	β-Bourbonene	$C_{15}H_{24}$	204	sesquiterpene					0.5	0.9												0.3		2.3						0.5	1.25																		
247	β-Caryophyllene	$C_{15}H_{24}$	204	sesquiterpene			7.6	3			0.2								1.22			9.1	1.1	6.5	0.2	0.1	1.8		29.8	9.9	20.79		3.3				3.5	4.41	2.25	2.8		6.6	4.2			0.1 ± 0.01	0.1		
248	β-Chamigrene	$C_{15}H_{24}$	204	sesquiterpene						0.9																																							
249	β-Copaene	$C_{15}H_{24}$	204	sesquiterpene					0.2	0.2												1.2		0.4						0.5						0.2													
250	β-Cubebene	$C_{15}H_{24}$	204	sesquiterpene					0.2													0.3							5.3		0.14																		
251	β-Elemene	$C_{15}H_{24}$	204	sesquiterpene			0.1		1.9	14.1								2.75	4.13			1.4		0.5						1.5	1.52		0.9			1.2		1.15	0.61		0.07			0.1					1.86
252	β-guaiene	$C_{15}H_{24}$	204	sesquiterpene																													0.4																
253	β-Santalene	$C_{15}H_{24}$	204	sesquiterpene																																0.5		0.29	0.23										
254	β-Selinene	$C_{15}H_{24}$	204	sesquiterpene			0.7	0.8		1.4								0.81				0.6		0.2			1.3				7.69					0.6		0.3	0.37			2.9		0.3					6.43
255	β-Sesquiphellandrene	$C_{15}H_{24}$	204	sesquiterpene														0.18				0.1									0.2												0.1						
256	β-Ylangene	$C_{15}H_{24}$	204	sesquiterpene						0.2																																							
257	γ-Cadinene	$C_{15}H_{24}$	204	sesquiterpene			1.8	0.7	0.2	0.4								0.31	1.97	0.27				0.3			0.7		2.2	0.4						1.1		1.24	1.66										1.12
258	γ-Curcumene	$C_{15}H_{24}$	204	sesquiterpene																																0.1													
259	γ-Elemene	$C_{15}H_{24}$	204	sesquiterpene															1.33					0.2														0.27	0.24										
260	γ-Humulene	$C_{15}H_{24}$	204	sesquiterpene																																												2.53	
261	γ-Muurolene	$C_{15}H_{24}$	204	sesquiterpene					0.6															0.2			0.2			0.7	5.82					1			0.44			0.5							
262	γ-patchoulene	$C_{15}H_{24}$	204	sesquiterpene			0.3	0.1																																									
263	δ-Amorphene	$C_{15}H_{24}$	204	sesquiterpene														0.32	1.88			0.1		0.2																									
264	δ-Cadinene	$C_{15}H_{24}$	204	sesquiterpene					1.7	1.2								0.49	0.43			0.9		1			1		5.6	6.7	0.3							1.18	1.89		1.32	2		0.2					2.15
265	δ-Elemene	$C_{15}H_{24}$	204	sesquiterpene					1.7										0.04					0.1					0.4	0.1			0.3			0.1													4.66
266	δ-Selinene	$C_{15}H_{24}$	204	sesquiterpene					6.2										3.11																														
267	ε-Amorphene	$C_{15}H_{24}$	204	sesquiterpene															0.58																														
268	eugenyl acetate	$C_{12}H_{14}O_3$	206	sesquiterpene																																									18.1				
269	Germacrene D	$C_{15}H_{26}$	206	sesquiterpene					6.9	0.4												7.3		6.1					4.2	2.3							2.5	3.16	3.14			3.7					0.2		14.89
270	Methyl Z3,Z6,E8-dodecatrienoate	$C_{11}H_{18}O_2$	208	monoterpene																																					0.36								
271	Geranyl butanoate	$C_{14}H_{24}O$	208	monoterpene																																								0.2					
272	Benzyl benzoate	$C_{14}H_{12}O_2$	212	aliphatic						0.1																																						40.9	
273	Tetradecanal	$C_{14}H_{28}O$	212	aliphatic																																								0.1					
274	epi-Cyclocolorenone	$C_{15}H_{22}O$	218	sesquiterpene					0.3																																								
275	8,9-dehydrothymyl isobutyrate	$C_{14}H_{18}O_2$	218	sesquiterpene																													5.9																
276	8-oxo-Neoisolongifolene	$C_{15}H_{22}O$	218	sesquiterpene															0.82																														
277	Dendrolasin	$C_{15}H_{22}O$	218	sesquiterpene																				0.3																									
278	Nootkatone	$C_{15}H_{22}O$	218	sesquiterpene															0.15																														
279	Silphiperfol-5-en-3-one B	$C_{15}H_{22}O$	218	sesquiterpene																													0.1																
280	Silphiperfol-6-en-5-one	$C_{15}H_{22}O$	218	sesquiterpene																													0.2																
281	trans-Calamenen-10-ol	$C_{15}H_{22}O$	218	sesquiterpene																																		1.72	2.49										
282	trans-α-Atlantone =[(E)-Atlantone]	$C_{15}H_{22}O$	218	sesquiterpene															0.57																														
283	7-Hydroxyfarnesene	$C_{15}H_{24}O$	220	sesquiterpene																		0.1																											
284	Eudesma-4(15),7-dien-1β-ol	$C_{15}H_{24}O$	220	sesquiterpene															4.94			0.1		1.3																									
285	Germacra-4(15),5,10(14)-trien-1α-ol -ol	$C_{15}H_{24}O$	220	sesquiterpene																		0.3																											
286	Thymyl isobutyrate	$C_{14}H_{20}O_2$	220	sesquiterpene																													13.9																
287	(5α)-Caryophylla-4(12),8(13)-dien-5-ol	$C_{15}H_{24}O$	220	sesquiterpene																		0.2											0.5			0.3													
288	(Z)-Caryophyllene oxide	$C_{15}H_{24}O$	220	sesquiterpene																		0.2														0.2													
289	(Z)-α-santalol	$C_{15}H_{24}O$	220	sesquiterpene			0.7	0.2																																									
290	14- Hydroxy-(Z)- caryophyllene	$C_{15}H_{24}O$	220	sesquiterpene																																							0.2						
291	14- Hydroxy-9- epi-(E)-caryophyllene	$C_{15}H_{24}O$	220	sesquiterpene			2.4		0.2																																		0.1						
292	14-Hydroxy-α-muurolene	$C_{15}H_{24}O$	220	sesquiterpene															0.83																														
293	14-Hydroxy-δ-cadinene	$C_{15}H_{24}O$	220	sesquiterpene															0.3																														
294	allo-spathulenol	$C_{15}H_{24}O$	220	sesquiterpene																													1.7																
295	Caryophylla-4(12),8(13)-dien-5α-ol	$C_{15}H_{24}O$	220	sesquiterpene																																							0.1						
296	Caryophylla-4(12),8(13)-dien-5β-ol	$C_{15}H_{24}O$	220	sesquiterpene																													0.4																
297	Caryophyllene epoxide =[Caryophyllene oxide]	$C_{15}H_{24}O$	220	sesquiterpene			18	6.9							5.64			0.65		0.59		0.1		3		1.8	2.8		6.3	16	1.18		0.4			3.7		1.6	1.27		0.66	2.6	1.6	1.9					
298	cedr-8(15)-en-9-α-ol	$C_{15}H_{24}O$	220	sesquiterpene						1.1																																							
299	Eudesma-4(15),7-dien-1β-ol	$C_{15}H_{24}O$	220	sesquiterpene																																													
300	Eudesma-4,11-dien-2-ol	$C_{15}H_{24}O$	220	sesquiterpene															0.66																														
301	Farnesal	$C_{15}H_{24}O$	220	sesquiterpene														0.27																															
302	Guaia-3,10(14)-dien-11-ol	$C_{15}H_{24}O$	220	sesquiterpene															4.52																														
303	Himachalene oxide	$C_{15}H_{24}O$	220	sesquiterpene															0.48																														
304	Humulene epoxide II	$C_{15}H_{24}O$	220	sesquiterpene			3.3	2.1			0.1													0.4	0.1	0.3	0.5			4.7						1.5		0.61					0.3						
305	Isospathulenol	$C_{15}H_{24}O$	220	sesquiterpene					1.1													0.4																											
306	Salvial-4(14)-en-1-one	$C_{15}H_{24}O$	220	sesquiterpene																		0.6		0.7						1.7																			
307	Silphiperfol-5-en-3-ol A	$C_{15}H_{24}O$	220	sesquiterpene																													0.3																
308	Silphiperfol-5-en-3-ol B	$C_{15}H_{24}O$	220	sesquiterpene																													0.2																
309	Spathulenol	$C_{15}H_{24}O$	220	sesquiterpene					4.7	0.2								0.6	3.74			2.3		1.3					5.1				0.9			1.3			0.93			2.1							
310	trans-14-Hydroxy-9-epi-β-caryophyllene	$C_{15}H_{24}O$	220	sesquiterpene															1.98																														
311	β-Bisabolenol	$C_{15}H_{24}O$	220	sesquiterpene															0.65																														
312	β-Caryophyllene oxide	$C_{15}H_{24}O$	220	sesquiterpene														0.58																															
313	β-copaen-4α-ol	$C_{15}H_{24}O$	220	sesquiterpene																													0.3																
314	γ-Costol	$C_{15}H_{24}O$	220	sesquiterpene															0.28																														
315	(2E,6E)-Farnesol	$C_{15}H_{26}O$	222	sesquiterpene																																													
316	Caryophylla-4(14),8(15)-dien-5α-ol	$C_{15}H_{26}O$	222	sesquiterpene																						0.2	0.7																						
317	α-Muurolol [= Torreyol]	$C_{15}H_{26}O$	222	sesquiterpene			3.3	0.1		0.3												0.4		1.9		0.4	1.8									0.5													
318	γ-Eudesmol	$C_{15}H_{26}O$	222	sesquiterpene														1.63						1.5			1.2			0.6																			

319	δ-Cadinol	C15H26O	222	sesquiterpene														3.18				0.3																											
320	Apiole	C12H14O4	222	sesquiterpene											1.98																																		
321	(4α)-Selin-11-en-4-ol	C15H26O	222	sesquiterpene																		0.3																											
322	(E)-3,7,11-Trimethyl-1,6,10-dodecatrien-3-ol = ((E)-Nerolidol)	C15H26O	222	sesquiterpene			5.4												0.94			16.6											0.7			0.2	1.5	0.37	1.53	0.6		4.9		0.1					
323	(Z)-Nerolidol	C15H26O	222	sesquiterpene														0.42				0.1																			1.11								
324	1,10-Di-epi-cubenol	C15H26O	222	sesquiterpene															2.54										1.9	2.6						0.4													
325	10-epi-γ-Eudesmol	C15H26O	222	sesquiterpene														6.83																															
326	1-epi-Cubenol	C15H26O	222	sesquiterpene						0.3									0.82										2.5							0.9													
327	γ-Eudesmol	C15H26O	222	sesquiterpene															0.09																														
328	5-epi-7-epi-α-Eudesmol	C15H26O	222	sesquiterpene																				0.4																									
329	7-epi-α-Eudesmol	C15H26O	222	sesquiterpene																				0.5																									
330	Bulnesol	C15H26O	222	sesquiterpene																							0.6																						
331	cis-Cadin-4-en-7-ol	C15H26O	222	sesquiterpene																				0.6																									
332	Cubeban-11-ol	C15H26O	222	sesquiterpene														0.22																															
333	Cubebol	C15H26O	222	sesquiterpene					0.4													1		0.4									0.3					0.75						2.6					
334	Drimenol	C15H26O	222	sesquiterpene															2.97																														
335	Elemol	C15H26O	222	sesquiterpene			1.5		0.3													0.1		0.7						0.3																	0.1		
336	epi-Cubebol	C15H26O	222	sesquiterpene																				0.4																		3.5		0.7					
337	epi-α-Bisabolol	C15H26O	222	sesquiterpene															0.59																														
338	epi-α-muurolol	C15H26O	222	sesquiterpene			1.2																																										
339	Eremoligenol	C15H26O	222	sesquiterpene																							1.1																						
340	Eudesmol	C15H26O	222	sesquiterpene																																													0.51
341	Germacren-D-4-ol	C15H26O	222	sesquiterpene														0.28	1.56			0.7																											
342	Gleenol	C15H26O	222	sesquiterpene																		0.1																											
343	Globulol	C15H26O	222	sesquiterpene					0.2																	2.4	5.8			1.05									1.5										
344	Guaiol	C15H26O	222	sesquiterpene				3.3	0.4		0.2							2.66	7.93						0.2	0.2	5.3			8.7																			
345	Hinesol	C15H26O	222	sesquiterpene			3.7	1.3										5.65																															
346	Intermedeol	C15H26O	222	sesquiterpene					0.7										0.74																														
347	Viridiflorol	C15H26O	222	sesquiterpene						0.2								0.17				0.2			7.9	24.2	47.6	7.76	5.7							14.5	7.4	12.97	18.8			4.4							
348	Isolongifolol	C15H26O	222	sesquiterpene															0.92																														
349	Ledol	C15H26O	222	sesquiterpene				1.4			0.8											0.5			0.8	0.2	0.7						0.4			0.9		0.71	1.21										
350	neo-Intermedeol	C15H26O	222	sesquiterpene					0.2																																								
351	Palustrol	C15H26O	222	sesquiterpene				4.4			0.1														0.1											0.8													
352	Selin-11-en-4α-ol	C15H26O	222	sesquiterpene															0.28																									51					
353	Selin-11-en-4β-ol	C15H26O	222	sesquiterpene																																								0.9					
354	Shyobunol	C15H26O	222	sesquiterpene																		0.1																0.8	1.38										
355	Valerianol	C15H26O	222	sesquiterpene														1.21																															
356	Viridiflorol	C15H26O	222	sesquiterpene			1	32.2			7.9								0.6																														
357	α-Bisabolol	C15H26O	222	sesquiterpene			0.4															0.1																						0.2					
358	α-Cadinol	C15H26O	222	sesquiterpene			0.9	3	1.5	2.4												0.6		1.3		0.2	0.6		1.7				0.3			1.9		1.98	3.13										
359	α-Elemol	C15H26O	222	sesquiterpene															1.56																														
360	α-Eudesmol	C15H26O	222	sesquiterpene							0.1														0.1	0.2	2.5																						
361	β-Cadin-4-en-10-ol	C15H26O	222	sesquiterpene														1.81																															
362	β-Eudesmol	C15H26O	222	sesquiterpene			8.9	1.4			0.2													11.4	0.1	0.2	2.7			0.4																			
363	τ-Cadinol	C15H26O	222	sesquiterpene							0.1														0.1				4.3							1.3		2.81	3.98										0.39
364	τ-Muurolol	C15H26O	222	sesquiterpene					0.6	1.2												0.2											0.2			0.9								0.2					
365	Cadin-4-en-10-ol =[α-cadinol]	C15H26O	222	sesquiterpene															3.96																														
366	Lavandulyl isobutyrate	C14H24O2	224	sesquiterpene																													0.1																
367	Neryl isobutyrate	C14H24O2	224	monoterpene																													1																
368	3-Methyl-5-(1,4,4-trimethylcyclohex-2-enyl) pentan-1-ol	C15H28O	224	aliphatic														2.9																															
369	Pentadecanal	C15H30O	226	aliphatic																		0.2																						0.9					
370	Pentadecanol	C15H32O	228	aliphatic																																												8.3	
371	4,6-Eudesmadiene-3,8-dione	C15H20O2	232	sesquiterpene															0.05																														
372	Polygodial	C15H22O2	234	sesquiterpene															1.25																														
373	Zerumbone epoxide	C15H22O2	234	sesquiterpene															0.06																														
374	Zizanoic acid	C15H22O2	234	sesquiterpene															0.18																														
375	Thymyl 2-methylbutanoate	C15H22O2	234	sesquiterpene																													1.6																
376	7-Hydroxyeudesm-4-en-6-one	C15H24O2	236	sesquiterpene															2.8																														
377	Geranyl tiglate	C15H24O2	236	sesquiterpene														1.36																															
378	trans-Humul-(9E)-en-2,6-dione	C15H24O2	236	sesquiterpene															1.1																														
379	Mint sulfide	C15H24S	236	sulfur compound					0.5																																								4.79
380	Agglomerone	C13H18O	238	sesquiterpene																																													
381	Neryl 2-methylbutanoate	C15H26O2	238	sesquiterpene																													1.1																
382	α-Bisabolol oxide B	C15H26O2	238	sesquiterpene																							0.4																						
383	Cryptomeridiol	C15H28O2	240	sesquiterpene															0.14																														
384	epi-Cryptomeridiol	C15H28O2	240	sesquiterpene															0.18																														
385	Heptadecane	C17H36	240	aliphatic																																					0.05								
386	Hexadecanol	C16H34O	242	aliphatic																																								0.1				11.9	
387	Fokienol	C17H28O	248	sesquiterpene																		0.1											0.8																
388	Flavesone	C14H20O4	252	sesquiterpene																																													
389	Palmitic acid	C16H32O2	256	aliphatic						0.2																																							
390	(5E,9E)-Farnesyl acetone	C18H30O	262	sesquiterpene					0.2																																								
391	cedr-8(15)-en-9-yl-acetate	C17H26O2	262	sesquiterpene						1.1																																							
392	6-methoxythymyl isobutyrate	C16H24O3	264	sesquiterpene																													0.9																
393	(2E,6Z)-Farnesyl acetate	C17H28O2	264	sesquiterpene																																													
394	Caryophyllene acetate	C17H28O2	264	sesquiterpene																																													
395	(Z)-Nerolidyl acetate	C17H28O2	264	sesquiterpene														0.34																															
396	Pimaradiene	C20H32	272	diterpene															0.55																														
397	Abietadiene	C20H32	272	diterpene															0.98																														
398	Kaurene	C20H32	272	diterpene															0.3																														
399	Neocembrene	C20H32	272	diterpene															0.09																														
400	p-Camphorene	C20H32	272	diterpene															0.12																														
401	Linalool anthranilate	C17H23NO2	273	amine																																												4.8	
402	Neophytadiene	C20H38	278	diterpene																																											0.1		
403	(E)-Isovalencenol	C20H34O	290	diterpene															0.34																														
404	(E,E)-Geranyl linalool	C20H34O	290	diterpene																		0.1																											
405	Geranylgeraniol	C20H34O	290	diterpene					9.1																																								
406	(E)-Phytol	C20H40O	297	diterpene			0.4															0.1		0.4																									
